# How Experience of Online Group Polarization Relates to University Students’ Value-Related Judgments and Choices: The Parallel Mediating Roles of Cognitive Dissonance and Emotional Contagion

**DOI:** 10.3390/bs16091697

**Published:** 2026-09-20

**Authors:** Shaosen He, Jiawei Li, Runcong Yang, Zhekun Wu, Huanhua Lu

**Affiliations:** 1 School of Marxism, China University of Geosciences Beijing, Beijing 100083, China; shaosenhe@cugb.edu.cn (S.H.);; 2 School of Economics and Management, China University of Geosciences Beijing, Beijing 100083, China; 3 School of Ocean Sciences, China University of Geosciences Beijing, Beijing 100083, China

**Keywords:** experience of online group polarization, university students, value judgment difficulty, value choice susceptibility, cognitive dissonance response, emotional contagion response, structural equation modeling

## Abstract

The psychological processes linking polarization experiences across different online interaction contexts to university students’ value-related judgments and choices warrant further investigation. This study distinguishes experienced polarization within online communities (EPC) from experienced polarization in open online discussions (EPD) and examines their associations with university students’ value-related judgments and choices, including parallel indirect associations through cognitive dissonance response (CDR) and emotional contagion response (ECR). Data from 2105 valid cross-sectional self-report questionnaires collected at a university in China were randomly divided into two subsamples: 770 cases for exploratory factor analysis and 1335 cases for confirmatory factor analysis and structural equation modeling. Indirect effects were estimated using 5000 bootstrap resamples. After adjustment for seven covariates covering demographics, internet use, and online participation, both forms of polarization experience were significantly and positively associated with CDR and ECR. Both psychological responses were also significantly and positively associated with value judgment difficulty (VJD) and value choice susceptibility (VCS). All four direct paths from the two forms of polarization experience to VJD and VCS were significant. Standardized estimates for the eight specific indirect effects ranged from 0.032 to 0.198, and all 95% bias-corrected confidence intervals excluded zero. Together, the predictors in the respective regression equations explained 44.4% of the variance in VJD and 54.5% in VCS. These findings provide statistical support for the hypothesized mediation relationships, indicating a pattern in which direct and indirect associations coexist. By distinguishing polarization experiences in specific interaction contexts, the study jointly connects cognitive and emotional responses to judgment formation and the reference points informing choices. It thereby provides empirical evidence for understanding how university students respond to online opinions and forms the basis for their evaluations and choices.

## 1. Introduction

Social media has become an important arena in which university students obtain public information, participate in issue-related discussions, and make sense of social reality (Alhabash & Ma, 2017). Platform affordances, algorithmic ranking, and the visibility of interaction metrics not only broaden the range of information available to users but may also reshape how they perceive and judge group norms and the distribution of public opinion.

Group polarization was originally used to describe the tendency for a group’s average attitude to shift further in the direction of its initial inclination following discussion (Moscovici & Zavalloni, 1969; Myers & Lamm, 1976). In online settings, anonymity, deindividuation, identity salience, and group norms have created new conditions for polarization (Spears et al., 1990; Sunstein, 2002; Lee, 2007). Cross-platform research shows that online echo chambers are characterized by information homogeneity and circulation within like-minded clusters, while differences in information distribution mechanisms and network structures across platforms shape the diversity of information users encounter (Bakshy et al., 2015; Cinelli et al., 2021). However, online information environments do not necessarily form closed echo chambers. Flaxman et al. (2016) found that social media and search channels may be associated with both ideological segregation and exposure to information across ideological divides. Examining individuals’ overall media use, Dubois and Blank (2018) questioned the prevalence of echo chambers and emphasized the importance of political interest and diversity in media use. Research on online group polarization therefore needs to distinguish homogeneity within specific interaction spaces from the degree of closure in individuals’ broader information environments, without assuming that all online communities constitute echo chambers. Depending on the issue and the conditions of interaction, clustering within online communities and homogeneous expression may intensify emotional conflict, narrow the space for moderate exchanges, and sharpen identity boundaries (Cai & Zhao, 2024; K. Li, 2023; H. Wu, 2021). Exposure to diverse opinions does not necessarily reduce polarization. Without appropriately designed interaction contexts, direct exposure to opposing views may activate group members’ identity defenses and reinforce their existing positions (Bail et al., 2018). Group emotions are also an important driver of group polarization (Jin et al., 2023). The relationship between social media and social outcomes cannot be reduced to a simple account of technological determinism; it is shaped jointly by platform governance, social contexts, individual psychology, and specific patterns of use (Lorenz-Spreen et al., 2023). Social media effects thus depend on more than users’ exposure behaviors. Individual psychological processes also play a key mediating role in how media environments translate into specific social outcomes (Valkenburg & Peter, 2013).

The university years represent an important period during which students’ values continue to develop and adjust. Within increasingly diverse information environments, university students must continually construct understandings of themselves, society, and public affairs. Exposure to online group polarization may increase the uncertainty students experience when interpreting social reality, dealing with value conflicts, and making value-related choices (Lin & Liu, 2022; Xu, 2021; Xu, 2022; Yan, 2024). Information sources, group norms, and individual psychological responses may jointly shape how young people make judgments and behavioral choices in online environments (Xia et al., 2025).

Building on this literature, the present study examines the polarization experiences university students report in relatively stable online communities and open online discussions, focusing on their associations with cognitive dissonance response, emotional contagion response, and value-related judgments and choices. To this end, a parallel mediation model is specified to test statistical indirect associations through the two psychological responses while retaining direct paths from both forms of polarization experience to value judgment difficulty and value choice susceptibility. The analysis focuses on students’ subjective experiences when encountering online opinions, particularly their difficulties in forming judgments and the tendency for online cues to influence the reference points they use for evaluation and choice. The scope of interpretation is limited to psychological responses and evaluative activities within specific contexts.

This study extends existing research on opinion exposure and online group polarization in two respects. First, it separately operationalizes experienced polarization within online communities and experienced polarization in open online discussions, taking into account both the interaction setting and the specific experiences it encompasses. Building on research concerned with the relationship between encountered opinions and individuals’ existing positions, this approach examines how individuals perceive the opinion environments of different interaction spaces. The distinction captures both contextual differences and the specific content of subjective experiences. Second, the study applies theoretical accounts of cognitive dissonance and emotional contagion to both judgment formation and the reference points informing choices. It examines how efforts to achieve cognitive consistency and emotional responses relate to students’ judgment difficulties and the influence of online cues on their evaluative reference points. By linking two forms of contextual experience and two psychological responses to concrete evaluative activities, the study provides empirical evidence on how online group polarization relates to the bases on which university students form judgments and the reference points they use when making choices.

## 2. Literature Review, Theoretical Framework, and Research Hypotheses

### 2.1. Two Contexts for Experiencing Online Group Polarization

Classical explanations of group polarization include informational influence, social comparison theory, and social identity theory. Informational influence accounts propose that discussions within homogeneous groups expose members to more arguments supporting their initial inclinations (Myers & Lamm, 1976). Social comparison theory emphasizes that members seeking group approval adjust how they express their views in line with group norms and adopt positions that embody those norms more strongly than the group’s average stance (Brown, 1965; Myers, 1978). Social identity theory further suggests that when a particular group identity becomes salient, individuals tend to understand themselves and others in terms of ingroup prototypes and accentuate differences between the ingroup and outgroups (Tajfel & Turner, 1979). Political communication research has a long tradition of examining like-minded exposure, selective exposure, and cross-cutting exposure. This literature focuses on how the positions expressed in the information individuals encounter or by their discussion partners relate to their own existing views. Garrett (2009) noted that seeking information that supports one’s views does not necessarily entail systematic avoidance of differing perspectives. Wojcieszak and Mutz (2009) examined political disagreement across different online discussion spaces and found that groups in which politics was not the central topic could also provide opportunities for cross-cutting exposure. These studies suggest that exposure to like-minded and differing views need not be mutually exclusive, nor should online communities automatically be regarded as environments closed to divergent opinions. Building on this work, the present study focuses on two interaction contexts—relatively stable online communities and open online discussions—and examines the polarization-related experiences individuals report within them.

Social media have changed the conditions under which group polarization occurs (Treem & Leonardi, 2013; Van Bavel et al., 2021; Lorenz-Spreen et al., 2023; Zhang et al., 2025). This study focuses on two interaction contexts. The first comprises relatively stable online communities, such as interest groups, fan groups, gaming communities, issue-oriented groups, identity-based communities, and study groups. Members interact over time around shared interests or identities, while platforms repeatedly present similar content based on users’ prior follows and interactions, strengthening identification and group norms. Polarization in these settings does not necessarily take the form of open conflict. It may instead involve the repeated circulation of homogeneous content, widespread support for views within the group, and collective defensiveness in response to criticism from outsiders. This study refers to these experiences as “experienced polarization within online communities” (EPC).

The second context comprises open discussion spaces, including comment sections, on-screen comment streams (danmu), repost threads, and discussions of trending topics. In these spaces, different positions are brought together within a single visual interface. Like counts, repost counts, and the ranking of popular comments provide visible cues to group endorsement, making opinions more likely to become polarized, labeled, and aligned with opposing camps. Irrational expression may also give a small number of extreme participants greater visibility, leading observers to overestimate group disagreement and the extremity of others’ views (Mak et al., 2024). This study refers to these experiences as “experienced polarization in open online discussions” (EPD).

The distinction between EPC and EPD rests on the interaction context and the subjective experiences within it. EPC encompasses experiences in relatively stable online communities, including community exposure, perceived homogeneity in content circulation, and discomfort and a tendency to defend the community when it is criticized. EPD focuses on features of open discussions, including divisions in opinion, emotional expression, and the perceived rationality of interactions. Whereas like-minded and cross-cutting exposure are distinguished by whether encountered opinions align with an individual’s existing position, this study differentiates experiences according to the interaction contexts in which they occur and their specific content. Disagreement can occur within online communities, while like-minded opinions may also cluster in open discussions. The two forms of experience therefore do not map one-to-one onto exposure to like-minded or differing views, and an individual may report high levels of both EPC and EPD. These two context-specific operational definitions provide a basis for examining associations with cognitive and emotional responses and value-related judgments and choices. Measurement is confined to individuals’ self-reported experiences and does not assess objective polarization at the platform level or pre- to post-discussion changes in attitudes.

### 2.2. Measuring University Students’ Value Judgments and Choices

Values are relatively stable beliefs or goals that transcend specific situations and guide evaluation and action (Rokeach, 1973; Schwartz, 1992). They are closely related to social structures and cultural contexts and operate through individual identity, self-understanding, and concrete action (Hitlin & Piliavin, 2004). Value-related judgments and choices involve individuals’ understanding of information, social situations, and the interests expressed by different parties.

The university years mark an important transition from socialization contexts characterized by relative dependence to broader engagement in public life. Students continually adjust their self-identities and value priorities through academic study, peer interactions, career planning, and public participation. Social media bring diverse information, group comparisons, and public expression into this process. Research suggests that online communities, recommendation algorithms, short-video narratives, and the public opinion environment influence how university students understand social issues, evaluate the public interest, and formulate life goals (Ai et al., 2023; Chen, 2020; R. Li, 2021; W. Wu, 2024; Turkle, 2011; Couldry & Hepp, 2017).

This study treats value judgment difficulty and value choice susceptibility as two descriptive outcome variables. Value judgment difficulty (VJD) refers to the subjective difficulties individuals report in assessing information veracity, evaluating differing accounts, and judging complex issues in online settings. Value choice susceptibility (VCS) refers to individuals’ reported tendency to be influenced by online discussions or related online cues in choices involving the public interest, agreement with opinion leaders, consideration of practical benefits, and evaluations of themselves and their lives. Conceptually, VJD overlaps substantially in content with difficulty in evaluating information. This study situates VJD within the cognitive conditions underlying value-related judgments and does not posit that the two are entirely independent constructs. VCS is related to susceptibility to social influence, with its measurement confined to the reference points used for evaluation and choice in the online contexts described above.

VJD describes subjective experiences during judgment formation rather than directly assessing whether judgments are correct. VCS describes the influence of online cues captured by the study’s items, without presuming that such influence is positive or negative. Higher scores on these variables indicate greater self-reported judgment difficulty or a stronger self-reported tendency to be influenced, respectively; they do not indicate inferior values. Likewise, lower scores cannot be interpreted directly as greater rationality, greater autonomy, or superior values.

### 2.3. Polarization Experiences and Cognitive Dissonance Response

Cognitive dissonance theory proposes that when individuals perceive inconsistency between related cognitions, they may experience psychological discomfort and become motivated to reduce that inconsistency and restore cognitive consistency (Festinger, 1957; Elliot & Devine, 1994). In online interactions, conflict between individuals’ existing judgments and dominant group opinions may manifest as discomfort and inner conflict, accompanied by doubts about their initial judgments and reconsideration of their understanding. Accordingly, this study treats cognitive dissonance as a theoretical mediator linking experience of online group polarization to value-related judgments and choices and operationalizes it as “cognitive dissonance response.”

Cognitive dissonance response (CDR) refers to university students’ reported tendencies to question their existing judgments, experience discomfort arising from discrepancies between their own views and group opinions, and reconsider or adjust their understanding when encountering majority opinions, dominant group views, or differing perspectives online. The term “response” emphasizes subjective manifestations associated with cognitive dissonance. It does not equate reconsideration itself with the degree of dissonance or assume that adjustments in understanding necessarily lead to negative outcomes.

Cognitive dissonance and informational influence may occur within the same online interaction, but their explanatory emphases differ. Informational influence concerns individuals’ use of others’ opinions as a basis for assessing reality, whereas cognitive dissonance theory focuses on discomfort arising from inconsistency and the motivation to restore consistency (Deutsch & Gerard, 1955; Festinger, 1957). This study places discomfort arising from inconsistency at the theoretical core of CDR and examines related responses alongside self-doubt and adjustments in understanding. CDR is therefore interpreted on the basis of the three items considered together, rather than treating self-doubt or reconsideration alone as evidence that cognitive dissonance has occurred. These cognitive manifestations may also contain elements of informational influence, and the current measures cannot fully distinguish the two processes.

In online communities, the repeated appearance of similar opinions reinforces the perception that “most members think this way.” When individuals’ judgments differ from the dominant views of a personally meaningful community, group consensus may make this discrepancy more salient, accompanied by discomfort and cognitive adjustment. Group polarization does not necessarily imply that individuals consistently endorse the group’s views. Rather, a highly consensual group environment may heighten awareness of group norms, making dissenting members more conscious of discrepancies between their own views and those of the group and thereby increasing cognitive strain (Cialdini & Goldstein, 2004). To maintain their group identity, individuals may question their existing judgments or engage in self-persuasion to align their thinking more closely with the group’s position. Accordingly, the following hypothesis is proposed:

**H1.**  *Experienced polarization within online communities is significantly and positively associated with cognitive dissonance response among university students*.

In open online discussions, university students encounter conflicting viewpoints, visible majority opinions, and rhetoric reflecting opposing group positions. These conflicting views may challenge existing judgments and give rise to experiences of inconsistency. Contradictory information makes factual judgments more difficult, while the ranking of popular comments, likes, and group attacks can generate substantial pressure from the prevailing climate of opinion. When individuals’ own views conflict with the visibly dominant position, they weigh whether to express those views, maintain their original judgments, or accept the majority opinion. Accordingly, the following hypothesis is proposed:

**H2.**  *Experienced polarization in open online discussions is significantly and positively associated with cognitive dissonance response among university students*.

Cognitive dissonance theory emphasizes the motivation to restore consistency but does not assume a particular direction of cognitive adjustment. In the online group polarization contexts considered here, when tension between personal judgments and group opinions remains unresolved, persistent discomfort and reassessment may coexist with difficulty forming definite judgments. Individuals may also rely more on external cues, such as group consensus or opinion leaders, to reconcile their own judgments with others’ views. Based on this contextual account, the study expects cognitive dissonance response to be positively associated with value judgment difficulty and value choice susceptibility. At the same time, adjustments in understanding may reflect careful reflection or cognitive updating. The hypotheses therefore concern overall associations between the variables and do not imply that all cognitive adjustments are negative. The following hypotheses are proposed:

**H5a.**  *Cognitive dissonance response is significantly and positively associated with value judgment difficulty among university students*.

**H5b.**  *Cognitive dissonance response is significantly and positively associated with value choice susceptibility among university students*.

### 2.4. Polarization Experiences and Emotional Contagion Response

Emotional contagion theory concerns the relationship between others’ emotional expressions and changes in an individual’s own emotions. Classical accounts emphasize processes such as mimicry, feedback, and emotional convergence (Hatfield et al., 1993). Measurement research also examines individual susceptibility to others’ emotions (Doherty, 1997). Although online communication lacks some of the nonverbal cues available in face-to-face interactions, text, symbols, images, and interaction feedback can also convey emotions. A large-scale Facebook experiment showed that reducing users’ exposure to positive or negative expressions changed the proportions of positive and negative emotion words in their subsequent posts (Kramer et al., 2014). This study treats emotional contagion as another theoretical mediator linking experience of online group polarization to value-related judgments and choices and examines its self-reported manifestations in online interactions through emotional contagion response (ECR). Emotional contagion response refers to university students’ reported tendencies to respond emotionally, be influenced by others’ emotions, and experience the continuation of those emotions into offline life when encountering online disputes and others’ emotional expressions. Being influenced by others’ emotions is central to the construct’s connection with emotional contagion theory. Emotional persistence provides a supplementary account of the duration of the response and is not itself equated with the process through which emotional contagion occurs.

Online group polarization is often accompanied by anger, sarcasm, anxiety, humiliation, and moral condemnation. Language conveying moral emotions spreads readily within ideological groups. Brady et al. (2017) found that each additional moral-emotional word in a social media message was associated with an approximately 20% increase in its expected retweet rate, with this pattern more pronounced within online groups. Subsequent research found that social feedback, such as likes and reposts, amplifies emotional expression and that users also adjust their emotional expression in response to the norms they observe online (Brady et al., 2021). These studies provide a basis for understanding the diffusion of emotional cues and the reinforcement of emotional expression online. However, the spread of emotional content and increases in emotional expression do not directly establish emotional contagion among recipients. This study therefore focuses on individuals’ reported responses to the influence of others’ emotions.

In relatively stable online communities, a shared identity makes members more likely to see issues of concern to the group as matters that “concern us” and to become more sensitive to the group’s emotional norms. Repeated exposure to members’ negative emotions, such as anger, feelings of being wronged, and anxiety, may be accompanied by individual emotional involvement. Accordingly, the following hypothesis is proposed:

**H3.**  *Experienced polarization within online communities is significantly and positively associated with emotional contagion response among university students*.

In open online discussions, emotional expression is more visible and confrontational. Intense language and moral condemnation are more likely to attract attention and engagement, whereas moderate expressions may be marginalized because they lack a similar advantage in diffusion. Even when observing rather than participating in discussions, university students may be affected by others’ expressions of anger, anxiety, and other emotions and experience corresponding emotional responses. Accordingly, the following hypothesis is proposed:

**H4.**  *Experienced polarization in open online discussions is significantly and positively associated with emotional contagion response among university students*.

Emotions influence not only subjective feelings but also processes of judgment and behavior. On social media, users’ emotional responses play an important role in linking the understanding of information to behavioral expression (Lu, 2024). In the online group polarization contexts examined here, individuals may report greater difficulty forming judgments when others’ emotional expressions elicit substantial emotional involvement and conflicting information remains unresolved. They may also rely more on cues from online groups, opinion leaders, and related discussions when making choices. Based on this contextual account, the study expects emotional contagion response to be positively associated with value judgment difficulty and value choice susceptibility. This expectation does not imply that emotional involvement necessarily reduces judgment quality, nor does it provide a basis for evaluating choices as better or worse in value terms. The following hypotheses are proposed:

**H6a.**  *Emotional contagion response is significantly and positively associated with value judgment difficulty among university students*.

**H6b.**  *Emotional contagion response is significantly and positively associated with value choice susceptibility among university students*.

### 2.5. Parallel Mediating Roles of Cognitive Dissonance Response and Emotional Contagion Response

Online group polarization does not directly and mechanically change individuals’ values simply because they encounter polarized information. Social identity theory and social comparison theory explain the role of conditions contributing to online group polarization, while cognitive dissonance theory and emotional contagion theory offer perspectives on individuals’ psychological responses in such contexts. Existing research suggests that cognitive appraisals and emotional responses often jointly influence information processing, attitude formation, and judgment when individuals encounter complex social information (Nabi, 1999). Recent research from a group psychology perspective also indicates that group behavior online is jointly influenced by emotional resonance, group identification, and psychological processing (Ni et al., 2026). In polarized environments, group norms, opinion pressure, and the emotional climate form the initial external context. The present study therefore expects related psychological responses to mediate the associations between polarization experiences and value-related judgments and choices.

This study treats cognitive dissonance and emotional contagion as two hypothesized mediating mechanisms. Cognitive dissonance theory explains the discomfort and cognitive adjustment associated with tension between personal judgments and online group opinions. Emotional contagion theory explains the relationship between others’ emotional expressions and individuals’ emotional responses. At the measurement level, CDR and ECR capture self-reported manifestations associated with these mechanisms. The analysis tests the theoretical expectation that the two forms of polarization experience are associated with value judgment difficulty and value choice susceptibility through these responses. The two mechanisms provide the theoretical basis for interpretation, while the questionnaire and structural equation model provide evidence of relationships among the corresponding variables and statistical indirect associations. On this basis, the two responses are specified as parallel rather than serial mediators. The following hypotheses are proposed:

**H7.**  *Cognitive dissonance response significantly mediates the associations of both forms of polarization experience with value judgment difficulty and value choice susceptibility*.

**H8.**  *Emotional contagion response significantly mediates the associations of both forms of polarization experience with value judgment difficulty and value choice susceptibility*.

**Figure 1:**
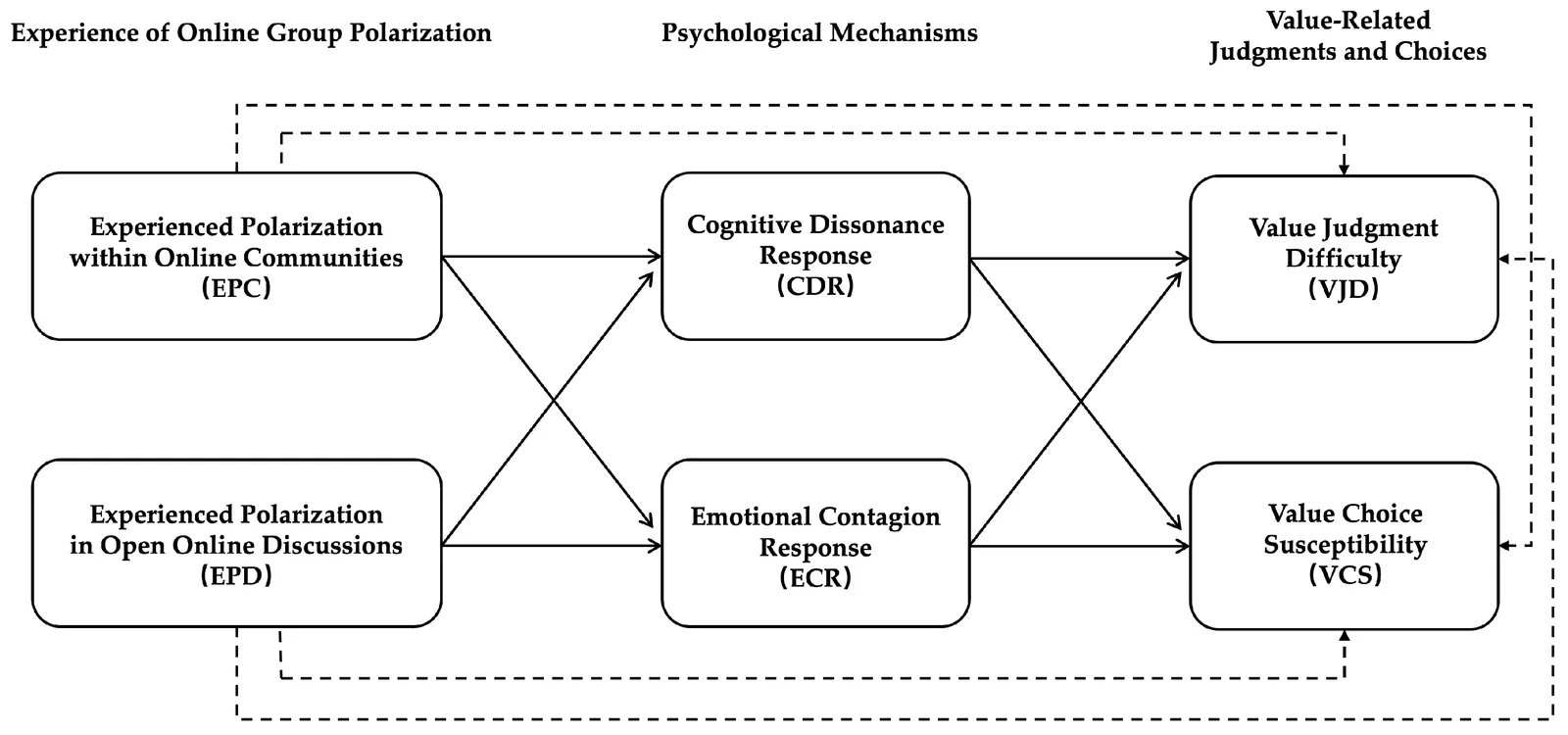
Parallel mediation model linking experience of online group polarization to university students’ value-related judgments and choices. Note: Solid lines represent hypothesized paths; dashed lines represent direct paths from polarization experiences to value judgment difficulty and value choice susceptibility.

Accordingly, the parallel mediation model proposed in this study is presented in Figure 1.

The main model retains direct paths from both forms of polarization experience to value judgment difficulty and value choice susceptibility and estimates specific indirect associations through cognitive dissonance response and emotional contagion response. This specification examines a statistical pattern in which direct and indirect associations coexist. The two psychological responses are treated as theoretically proposed mediating mechanisms, while their temporal ordering requires further examination through research design and tests of alternative models.

## 3. Materials and Methods

### 3.1. Participants and Data Collection

An online survey was conducted in June 2026 through Wenjuanxing among undergraduate and postgraduate students enrolled at China University of Geosciences (Beijing). The questionnaire was distributed through university-based online channels, including class groups, course groups, student organizations, and research groups. Each account could submit only one response. Given the exploratory nature of the study and the difficulty of obtaining a probability sample of internet users more broadly, online convenience sampling subject to stratified quotas was adopted. Distribution focused on balancing the sample across years of study, while demographic and online behavioral information was collected on gender, field of study, daily internet use, participation in online communities, and engagement in open online discussions. The planned quotas were 800 respondents in the first and second undergraduate years, 400 in the third and fourth undergraduate years, and 400 master’s and doctoral students.

A total of 2756 questionnaires were collected. Under the quality-control rules, 463 responses were excluded for failing the attention check, 44 for completion times below 60 s despite passing the attention check, and 144 for evident straightlining or logical inconsistencies. The final sample comprised 2105 valid responses, yielding a valid response rate of 76.38%. Of these respondents, 1062 were male (50.45%) and 1043 were female (49.55%). The sample included 699 first-year undergraduates (33.21%), 505 second-year undergraduates (24.00%), 280 third-year undergraduates (13.30%), 197 fourth-year undergraduates (9.36%), 312 master’s students (14.82%), and 112 doctoral students (5.32%). By field of study, 1523 respondents were in science and engineering (72.35%), 235 in economics and management (11.16%), 195 in the literature, history, and philosophy (9.26%), 95 in arts and physical education (4.51%), and 57 in education and other fields (2.71%).

The anonymous online questionnaire collected only demographic information and experiences of internet use relevant to the study objectives. No direct identifiers, such as names, contact details, or student identification numbers, were collected. The study involved no experimental intervention or potential physical risk, and technical information collected by the platform was not included in the analytical dataset. The questionnaire’s opening page explained the study purpose, anonymity, voluntary participation, and intended use of the data. Participants entered the main questionnaire only after reading this information and voluntarily choosing to continue, which was taken as indicating informed consent.

Data were collected using online stratified quota sampling rather than strict probability sampling. The sample therefore does not provide a representative basis for statistical inference to the entire population of university students in China.

### 3.2. Scale Development and Variable Measurement

The questionnaire contained 34 questions, including 24 core scale items, with Q19 serving as an attention check. All core variables were measured on five-point Likert scales, ranging from 1 (strongly disagree/does not describe me at all) to 5 (strongly agree/describes me very well). Initial items were generated from the relevant theoretical constructs. Three experts in psychology, youth values, and online communication reviewed the initial questionnaire, after which two pilot surveys were conducted. The first pilot survey (*N* = 129) identified inadequate internal consistency in some dimensions (Cronbach’s α = 0.166–0.465). The seven preliminary dimensions were consequently revised into six constructs comprising 24 core items. Item analysis in the second pilot survey (*N* = 65; overall α = 0.7643; KMO = 0.584) further identified some reverse-worded items, items combining multiple meanings, and items with unclear construct assignments. Drawing on expert feedback, the research team reduced such items and refined their wording, online contexts, and construct assignments. Six latent variables were ultimately retained: experienced polarization within online communities, experienced polarization in open online discussions, cognitive dissonance response, emotional contagion response, value judgment difficulty, and value choice susceptibility.

After the survey had been administered, five experts in fields related to psychology, youth values, and online communication who had not participated in the earlier questionnaire design conducted a semi-blind assessment of the content relevance and construct assignment of the 24 core items. They received the research topic, definitions of the six constructs, and items presented in randomized order with new item numbers. The researchers’ intended construct assignments were withheld. The experts rated each item’s relevance and assigned it to a construct. The item-level content validity index (I-CVI) was calculated as the proportion of experts assigning a relevance score of 3 or 4. The construct-matching rate was calculated as the proportion whose assignments agreed with the researchers’ intended construct. The matching rates for Q25, Q27, Q28, Q32, and Q33 were 20%, 0%, 0%, 20%, and 60%, respectively, all below the 80% screening threshold adopted for construct assignment. Four experts assigned Q25 to emotional contagion response, and all five assigned Q27 to cognitive dissonance response, indicating discrepancies between the content of these items and their originally intended outcome constructs. Based on these expert assignments and an analysis of item content, the five items were excluded, leaving 19 items. Recalculation using the same round of expert ratings yielded I-CVI values, a scale-level average content validity index (S-CVI/Ave), and a scale-level universal agreement content validity index (S-CVI/UA) of 1.000 for the retained items. Construct-matching rates ranged from 80% to 100%: Q15, Q18, Q29, and Q34 each achieved 80%, while the remaining 15 items achieved 100%. The overall matching rate was 95.79% (91/95), and Fleiss’ κ for agreement on construct assignment was 0.899. These expert ratings supported the content relevance of the retained items and their proposed construct assignments within the present study. The final assignments were Q10–Q12 for experienced polarization within online communities; Q13–Q15 for experienced polarization in open online discussions; Q16–Q18 for cognitive dissonance response; Q20–Q22 for emotional contagion response; Q23, Q24, and Q26 for value judgment difficulty; and Q29, Q30, Q31, and Q34 for value choice susceptibility. Q25, Q27, Q28, Q32, and Q33 were excluded from the primary scale scoring (Table 1 and Table A1).

The EPC items address exposure to online communities (Q10), perceived homogeneity in content circulation (Q11), and discomfort and a tendency to defend the community when it is criticized (Q12). The measure therefore encompasses both perceptions of the community context and individual involvement. Q10 does not directly indicate the degree of polarization, and Q12 is not interpreted as an objective measure of exposure behavior. The EPD items focus on respondents’ observations of divisions in opinion (Q13), others’ emotional expression (Q14), and rationality and opposing positions in discussions (reverse-scored Q15). They do not directly measure extremity in respondents’ own behavior. Scores for both constructs are interpreted in terms of the contextualized experiences covered by their respective items.

The CDR items were designed to capture subjective responses associated with cognitive dissonance. Q16 concerns doubts about existing judgments in the face of online majority opinions; Q17 directly addresses feeling “uncomfortable and conflicted” when personal views differ from the group’s dominant position; and Q18 concerns the tendency to reconsider and adjust one’s understanding when encountering different perspectives. The three items respectively address wavering judgments, discomfort arising from inconsistency, and adjustments in understanding. Of these, Q17 corresponds most directly to the psychological discomfort specified in cognitive dissonance theory. Including all three items in CDR is intended to capture related response tendencies in online opinion contexts. They are not treated as an observed sequence of stages, nor are the adjustments in understanding addressed by Q18 assumed to represent negative change. CDR concerns how individuals’ existing judgments and understanding respond to online opinions, whereas VJD concerns subjective difficulty forming judgments about information veracity, differing accounts, and complex issues. Reconsidering one’s understanding does not necessarily imply an inability to form a judgment, and judgment difficulty need not involve conflict between personal views and group opinions. These distinctions clarify the different aspects addressed by the items, but they do not rule out a shared component of cognitive uncertainty.

The ECR items were designed to capture the influence of online emotions and the responses associated with that influence. Q20 concerns negative emotional responses during online arguments; Q21 concerns the self-reported tendency for one’s emotions to be influenced by others’ emotions; and Q22 concerns the persistence of negative moods associated with online arguments into offline life. Q22 was retained to capture the persistence of emotional responses, with the qualification that it represents a subsequent manifestation and cannot independently identify the process of emotional contagion. ECR is interpreted in terms of the content jointly represented by the three items, encompassing emotional responses, the tendency to be influenced, and the persistence of related emotions. Because Q20 and Q22 cannot independently distinguish responses to others’ emotions from general emotional reactions to the content of an argument, ECR is treated as a self-report measure of responses associated with emotional contagion. Its findings alone cannot rule out alternative explanations, including general emotional reactivity.

Among the items retained for VJD and VCS, the “public interest” in Q29 is a reference point for choice specified in the item. The item captures respondents’ subjective experiences of the representativeness of online discussions and of making choices in that context. It is not used to evaluate their ability to analyze the public interest or their moral character. Nor does it directly measure conformity to online opinions or establish that actual choices have changed. The expressions “a sense of ease,” “involution,” and “lying flat” in Q34 are examples of online expressions. The item asks whether such expressions influence evaluations of oneself and one’s life, without assuming that using, endorsing, or being influenced by them constitutes “value deviation.” Although Q31 specifies the direction of “placing greater emphasis on practical benefits,” this is not interpreted as evidence of a deterioration in values.

The questionnaire was administered in Chinese, and the English items were used only for presentation in the manuscript and appendix (Appendix A). A supplementary translation–back-translation check was conducted after survey administration. Two bilingual researchers with social science backgrounds independently translated the items into English and reconciled their translations with the research team. Two other bilingual researchers who had neither participated in the forward translation nor seen the original Chinese items independently back-translated the reconciled version. The research team compared the versions item by item and resolved differences in meaning, tone, and culturally specific expressions. The check identified no substantive differences in the revised translation that altered the direction or core meaning of the original items.

### 3.3. Data Analysis

Data were analyzed using SPSS 27.0 and Mplus 8.3. After quality screening, the 2105 valid responses were randomly divided into two nonoverlapping subsamples: 770 cases for exploratory factor analysis and 1335 cases for descriptive statistics, correlation analysis, confirmatory factor analysis, and structural model testing. For the exploratory analysis, data suitability was first assessed using the Kaiser–Meyer–Olkin (KMO) measure and Bartlett’s test of sphericity. Common factors were then extracted using principal axis factoring with Kaiser-normalized oblique rotation. Item structure was examined using the criterion of initial eigenvalues greater than 1 and the pattern matrix. Descriptive statistics included the means and standard deviations of construct scores, and Pearson correlations were used to describe bivariate associations between variables.

Measurement model analysis was based on the 19 retained items. The six constructs were specified as first-order latent variables, and the items were treated as approximately continuous. Confirmatory factor analysis was conducted using robust maximum likelihood estimation (MLR). Five competing measurement models that combined different constructs were also specified, and their fit was compared to assess the appropriateness of the six-factor structure. Cronbach’s α and composite reliability (CR) were used to assess the internal consistency of each construct, and average variance extracted (AVE) was used to examine convergent validity. These analyses assessed internal consistency, convergent validity, and factor structure as distinct aspects of measurement quality in the present sample.

Model fit was evaluated jointly using χ^2^ and its degrees of freedom, χ^2^/df, CFI, TLI, RMSEA and its 90% confidence interval, and SRMR. Judgments considered the different indices alongside the model specification, rather than relying on a single indicator (Hu & Bentler, 1999; Kline, 2016). In addition, an unrotated principal component analysis of the core items was conducted for Harman’s single-factor test to examine the proportion of total variance explained by a single factor. This test served only as a preliminary diagnostic for common method bias.

The structural model was estimated using maximum likelihood (ML), with EPC and EPD specified as the focal latent predictors, CDR and ECR as parallel mediators, and VJD and VCS as endogenous outcome variables. Paths from EPC and EPD to both psychological responses, paths from both responses to VJD and VCS, and the four direct paths from EPC and EPD to VJD and VCS were estimated simultaneously. Gender (Q1), academic year (Q2), field of study (Q4), average daily internet use (Q5), online community membership status (Q7), frequency of browsing discussions of trending topics (Q8), and mode of participation in discussions of trending topics (Q9) were included as covariates in the regression equations for all six latent variables: EPC, EPD, CDR, ECR, VJD, and VCS. Gender (Q1) was coded as 0 for male and 1 for female, and field of study (Q4) as 0 for science and engineering and 1 for other fields. Academic year (Q2) was coded from 1 to 5, corresponding to the first through fourth undergraduate years and master’s or doctoral study, respectively. Average daily internet use (Q5), online community membership status (Q7), discussion-space browsing frequency (Q8), and discussion participation mode (Q9) were each coded from 1 to 5. Higher scores indicated longer internet use, more active community participation, more frequent browsing, and more active participation in discussions, respectively. The model allowed EPC and EPD to correlate and separately estimated the residual correlations between CDR and ECR and between VJD and VCS.

Path analyses reported STDYX-standardized coefficients with their corresponding standard errors, z values, and two-sided *p* values. The eight specific indirect effects through CDR and ECR were tested using 5000 bootstrap resamples. Standardized indirect effects and bias-corrected 95% confidence intervals were reported, with the exclusion of zero from the confidence interval serving as the primary criterion for statistical significance. R^2^ values for each endogenous latent variable were obtained directly from the same structural model output to describe the proportion of variance jointly explained by all predictors in the corresponding regression equation. Given the cross-sectional design, direct paths and indirect effects were interpreted as statistical associations conditional on the specified model and covariates. They were not used to establish the temporal order of psychological processes or confirm causal relationships.

## 4. Results

### 4.1. Exploratory Factor Analysis

Exploratory factor analysis was conducted on the 19 items in the exploratory subsample using SPSS 27.0. The KMO value was 0.881, and Bartlett’s test of sphericity was statistically significant (χ^2^ = 5422.278, df = 171, *p* < 0.001), indicating that the correlation structure among the items was suitable for factor analysis. Because the constructs could be correlated, common factors were extracted using principal axis factoring with oblique rotation and Kaiser normalization.

Six factors were extracted. The first six initial eigenvalues ranged from 1.039 to 6.065, and the cumulative variance explained after extraction was 54.513%. Extracted communalities ranged from 0.337 to 0.714. Factor extraction was completed after 18 iterations, and rotation converged after 10 iterations.

The pattern matrix showed that all 19 items had their largest absolute loadings on their hypothesized factors. Absolute primary loadings ranged from 0.521 to 0.860, all exceeding 0.50. The corresponding ranges for EPC, EPD, CDR, ECR, VJD, and VCS were 0.540–0.855, 0.594–0.750, 0.580–0.770, 0.709–0.801, 0.550–0.860, and 0.521–0.664, respectively. No absolute loading on a non-target factor exceeded 0.179, and the difference between the absolute primary loading and the largest absolute secondary loading was at least 0.348 for every item, indicating relatively clear item–factor assignments. The correlation coefficients among the six factors range from 0.040 to 0.577, and the factor correlation matrix is shown in Table 2.

### 4.2. Descriptive Statistics and Correlation Analysis

Descriptive statistics and Pearson correlations were calculated using the 1335 valid cases, as reported in Table 3. The mean scores for experienced polarization within online communities (EPC) and experienced polarization in open online discussions (EPD) were 3.46 (SD = 0.78) and 3.48 (SD = 0.79), respectively. The corresponding means for cognitive dissonance response (CDR) and emotional contagion response (ECR) were 2.93 (SD = 0.79) and 2.89 (SD = 0.98). Value judgment difficulty (VJD) and value choice susceptibility (VCS) had mean scores of 3.41 (SD = 0.85) and 3.06 (SD = 0.67), respectively.

Bivariate analyses revealed significant positive correlations among all six core constructs (r = 0.285–0.530, all *p* < 0.01). Both forms of experience of online group polarization were positively correlated with cognitive dissonance response and emotional contagion response (r = 0.285–0.420), as well as with value judgment difficulty and value choice susceptibility (r = 0.320–0.408). Cognitive dissonance response correlated with value judgment difficulty and value choice susceptibility at 0.430 and 0.495, respectively, while the corresponding correlations for emotional contagion response were 0.426 and 0.480. The correlation between the two forms of polarization experience was 0.368, that between the two psychological responses was 0.530, and that between value judgment difficulty and value choice susceptibility was 0.490.

Regarding internet use and online participation, average daily internet use (Q5) was significantly and positively correlated with all six core constructs (r = 0.127–0.183, all *p* < 0.01). Online community membership status (Q7), frequency of browsing discussions of trending topics (Q8), and mode of participation in discussions of trending topics (Q9) were also significantly and positively correlated with all six constructs. Their respective correlation ranges were 0.089–0.360, 0.170–0.325, and 0.101–0.360 (all *p* < 0.01). Given the coding of these variables, the results indicate that longer internet use, higher levels of community membership and activity, more frequent browsing of discussion spaces, and more active modes of discussion participation were associated with higher scores on both forms of polarization experience, both psychological responses, value judgment difficulty, and value choice susceptibility.

Among the demographic variables, gender (male = 0, female = 1) was significantly and positively correlated with EPC, ECR, and VJD, and significantly and negatively correlated with EPD. This indicates that female students had higher mean scores on the first three constructs and lower mean scores on EPD. Academic year codes were significantly and negatively correlated with EPD, CDR, and VJD, with coefficients of −0.056, −0.106, and −0.070, respectively (all *p* < 0.05). Field of study (science and engineering = 0, other fields = 1) was significantly and positively correlated with EPC, CDR, and ECR, with coefficients of 0.097, 0.065, and 0.071, respectively (all *p* < 0.05). The absolute correlations between these demographic variables and the core constructs did not exceed 0.168. All seven covariates were included simultaneously in the subsequent structural model to examine conditional associations among the core constructs.

### 4.3. Reliability and Validity Tests

Cronbach’s α and composite reliability (CR) were used to assess the internal consistency of each construct, and average variance extracted (AVE) was used to evaluate convergent validity. As shown in Table 4, Cronbach’s α ranged from 0.710 to 0.841 across the six constructs, and CR ranged from 0.711 to 0.841. All values exceeded the commonly used benchmark of 0.70, indicating generally acceptable internal consistency. AVE ranged from 0.406 to 0.638. ECR and VJD had AVE values above 0.50, whereas the remaining constructs had values between 0.406 and 0.495. Previous scale validation studies have adopted an AVE threshold of 0.40 as a more lenient criterion when composite reliability is acceptable (Jiang et al., 2025; Zhou et al., 2026). Nevertheless, convergent validity remains relatively modest for these four constructs.

### 4.4. Harman’s Single-Factor Test

All variables were measured using a self-report questionnaire administered at a single time point, creating a potential risk of common method bias (CMB). Harman’s single-factor test was used as an initial diagnostic assessment. The unrotated principal component analysis showed that the first factor explained 33.762% of the variance. No single factor accounted for most of the measurement variance. These results indicate no clear dominance of a single factor in the data. However, given the limitations of Harman’s single-factor test, common method bias cannot be ruled out entirely (Podsakoff et al., 2003). Confirmatory factor analysis was therefore also used to further assess the discriminant validity of the measurement model.

### 4.5. Confirmatory Factor Analysis

Confirmatory factor analysis (CFA) was conducted to further assess the structural validity of the measurement model. Consistent with the theoretical model, EPC, EPD, CDR, ECR, VJD, and VCS were specified as six first-order latent variables, and parameters were estimated using robust maximum likelihood (MLR).

The CFA results indicated that the six-factor measurement model fitted the data well: χ^2^ = 509.029, df = 137, χ^2^/df = 3.72, CFI = 0.951, TLI = 0.939, SRMR = 0.042, and RMSEA = 0.045, with a 90% confidence interval of [0.041, 0.049]. All indices met the recommended criteria for structural equation modeling. The good overall fit indicates that the theoretically specified six-factor measurement structure adequately represented the covariance relationships among the observed variables.

To further assess the superiority of the six-factor measurement structure, five alternative models were specified for comparison (Table 5). Model 2 combined EPC and EPD into a single “experience of online group polarization” factor. Model 3 additionally combined the mediators, CDR and ECR, and Model 4 further combined the outcome variables, VJD and VCS. Model 5 combined EPC, EPD, CDR, and ECR into one factor and VJD and VCS into another. Model 6 loaded all items onto a single latent variable.

The six-factor model demonstrated the best fit among the competing models, with the highest CFI and TLI and the lowest RMSEA and SRMR, indicating clearly better fit than the alternatives. These findings support the six-factor structure in the present sample, while the AVE values below 0.50 for four constructs indicate that convergent validity remains relatively modest (Table 4).

### 4.6. Structural Model and Hypothesis Testing

The structural model was estimated using maximum likelihood in Mplus 8.3 with the 1335 valid cases. It simultaneously included paths from EPC and EPD to CDR and ECR, paths from CDR and ECR to VJD and VCS, and four direct paths from EPC and EPD to VJD and VCS. Gender, academic year, field of study, average daily internet use, online community membership status, frequency of browsing discussions of trending topics, and mode of participation in discussions of trending topics were included as covariates in the regression equations for all six latent variables. Residuals were allowed to correlate between EPC and EPD, between CDR and ECR, and between VJD and VCS.

The structural model yielded the following fit indices: χ^2^ = 801.874, df = 228, χ^2^/df = 3.52, *p* < 0.001, CFI = 0.940, TLI = 0.920, RMSEA = 0.043, with a 90% confidence interval of [0.040, 0.047], and SRMR = 0.036. Taken together, these indices indicated acceptable overall fit, with RMSEA and SRMR indicating good fit. In addition, AIC was 62,491.033 and BIC was 63,083.455.

**Figure 2:**
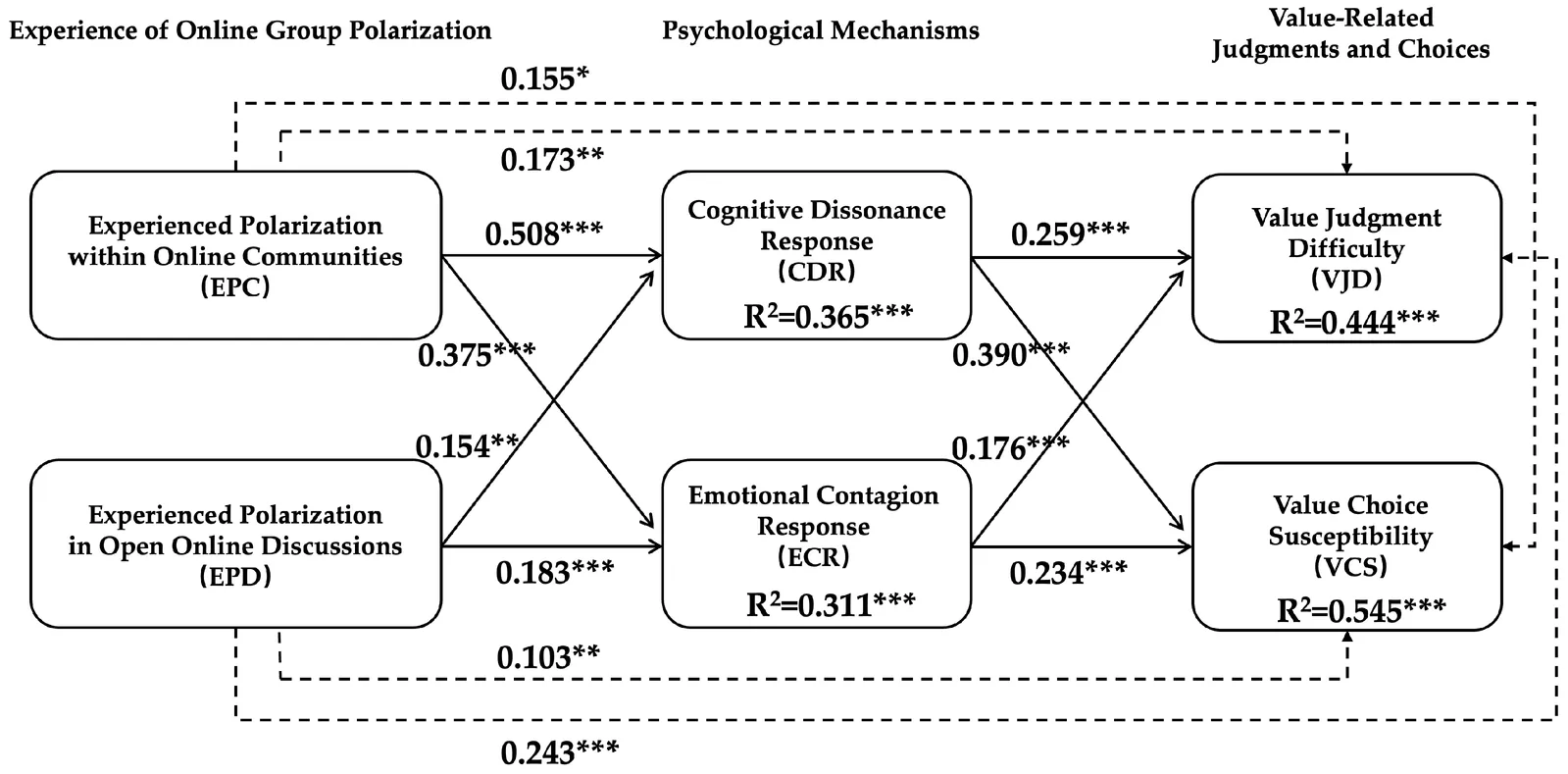
Structural equation model and standardized path coefficients. Note: Solid lines represent hypothesized paths; dashed lines represent direct paths from polarization experiences to value judgment difficulty and value choice susceptibility; * *p* < 0.05, ** *p* < 0.01, *** *p* < 0.001.

As shown in Figure 2 and Table 6, after adjustment for the seven covariates, EPC was significantly and positively associated with CDR (β = 0.508, *p* < 0.001), as was EPD (β = 0.154, *p* = 0.001), providing statistical support for H1 and H2. EPC and EPD were also significantly and positively associated with ECR, with standardized path coefficients of 0.375 and 0.183, respectively (both *p* < 0.001), supporting H3 and H4.

Regarding the associations between the two psychological responses and value-related judgments and choices, CDR was significantly and positively associated with both VJD (β = 0.259, *p* < 0.001) and VCS (β = 0.390, *p* < 0.001), supporting H5a and H5b. ECR was also significantly and positively associated with VJD (β = 0.176, *p* = 0.001) and VCS (β = 0.234, *p* < 0.001), supporting H6a and H6b. Thus, when both forms of polarization experience and the covariates were considered simultaneously, cognitive dissonance response and emotional contagion response were positively associated with respondents’ reported value judgment difficulty and value choice susceptibility.

The regression coefficients for the seven covariates are presented in Table 7. The model also retained direct paths from both forms of polarization experience to value-related judgments and choices. After accounting for CDR, ECR, and the covariates, EPC remained significantly and positively associated with VJD (β = 0.173, *p* = 0.007) and VCS (β = 0.155, *p* = 0.012). The direct associations of EPD with VJD (β = 0.243, *p* < 0.001) and VCS (β = 0.103, *p* = 0.010) were also statistically significant. The covariate results showed that average daily internet use, frequency of browsing discussions of trending topics, and mode of participation in discussions of trending topics were significantly and positively associated with both forms of polarization experience. Online community membership status was significantly and positively associated with EPC. Gender was positively associated with EPC and negatively associated with EPD, while academic year codes were negatively associated with EPD and CDR. In the VJD equation, after adjustment for the other predictors, average daily internet use was positively associated with VJD (β = 0.058, *p* = 0.024), whereas online community membership status and mode of participation in discussions of trending topics were negatively associated with VJD (β = −0.082, *p* = 0.007; β = −0.096, *p* = 0.002). None of the conditional associations between field of study and the six latent variables was statistically significant. Likewise, none of the seven covariate coefficients in the ECR and VCS equations reached statistical significance.

The R^2^ values for CDR, ECR, VJD, and VCS were 0.365, 0.311, 0.444, and 0.545, respectively. Thus, all predictors in the respective regression equations jointly explained 36.5%, 31.1%, 44.4%, and 54.5% of the variance in these latent variables. The explained variance in CDR and ECR reflects the joint contributions of EPC, EPD, and the seven covariates, whereas the explained variance in VJD and VCS reflects the joint contributions of EPC, EPD, CDR, ECR, and the seven covariates. In addition, the seven covariates explained 35.0% and 9.1% of the variance in EPC and EPD, respectively.

After accounting for EPC, EPD, and the seven covariates, CDR and ECR still showed a significant positive residual correlation (r = 0.556, S.E. = 0.042, z = 13.347, *p* < 0.001). After accounting for EPC, EPD, CDR, ECR, and the seven covariates, the residual correlation between VJD and VCS was also positive and statistically significant (r = 0.359, S.E. = 0.054, z = 6.648, *p* < 0.001). These values were taken from the corresponding WITH parameters in the STDYX-standardized results for the final model and describe correlations between the portions left unexplained by the respective regression equations.

### 4.7. Mediation Effect Analysis

Specific indirect effects were tested using 5000 bootstrap resamples in the parallel mediation model, which simultaneously included CDR, ECR, the seven covariates, and the four direct paths. STDYX-standardized indirect effects and their bias-corrected 95% confidence intervals are reported in Table 8. Whether the confidence interval excluded zero was the primary criterion for determining the statistical significance of an indirect effect.

For the paths through cognitive dissonance response, the standardized indirect effects of experienced polarization within online communities on value judgment difficulty and value choice susceptibility through CDR were 0.132 (95% CI [0.069, 0.211]) and 0.198 (95% CI [0.128, 0.291]), respectively. The corresponding standardized indirect effects of experienced polarization in open online discussions through CDR were 0.040 (95% CI [0.015, 0.076]) and 0.060 (95% CI [0.022, 0.109]). All four specific indirect effects were positive, and their confidence intervals excluded zero, providing statistical support for H7.

For the paths through emotional contagion response, the standardized indirect effects of experienced polarization within online communities on value judgment difficulty and value choice susceptibility through ECR were 0.066 (95% CI [0.026, 0.119]) and 0.088 (95% CI [0.043, 0.149]), respectively. The corresponding standardized indirect effects of experienced polarization in open online discussions through ECR were 0.032 (95% CI [0.013, 0.063]) and 0.043 (95% CI [0.019, 0.078]). All four specific indirect effects were also positive, and their confidence intervals excluded zero, providing statistical support for H8.

Taken together, the structural path and indirect-effect analyses showed that all four direct associations between the two forms of experience of online group polarization and value-related judgments and choices were significant. All eight specific indirect associations through cognitive dissonance response and emotional contagion response were also supported. This statistical pattern, in which direct and indirect associations coexist, provides empirical support for the mediating roles of the two psychological responses in the relationships of experience of online group polarization with value judgment difficulty and value choice susceptibility. Given the cross-sectional design, these mediation effects should be interpreted as statistical indirect associations under the current model specification. The temporal ordering and causal direction of the relationships require further examination.

In supplementary analyses, the two serial mediation models, CDR → ECR and ECR → CDR, both yielded the same overall fit as the parallel model. This result is consistent with covariance equivalence under the current specification. Model fit therefore cannot distinguish the temporal ordering of the two responses.

## 5. Discussion

### 5.1. Contextual Differences in Polarization Experiences and Individual Cognitive Tension

This study examined polarization experiences in online communities and open discussions separately. When both forms of experience were included in the same model, along with seven covariates, each remained significantly and positively associated with cognitive dissonance response and emotional contagion response. This finding indicates that understanding how students participate in online interactions should be complemented by attention to how they perceive the opinion environments within those interactions. The study thus adds a perspective to existing research on opinion exposure by linking subjective experiences in different interaction contexts to psychological responses when individuals encounter disagreement.

The positive association between EPC and CDR warrants particular consideration. EPC was significantly and positively associated with CDR (β = 0.508, *p* < 0.001), and the standardized indirect effect linking EPC to VJD through CDR was 0.132, with a 95% bias-corrected confidence interval of [0.069, 0.211]. This association can be understood in terms of the dual role of group opinions: they provide accounts of events while also serving as social reference points against which members evaluate their own understanding. When personal judgments differ from the opinions of a group to which students feel they belong, students must also consider why their understanding differs from that of the group. Matz and Wood’s (2005) research on disagreement within groups provides a basis for understanding the cognitive tension accompanying such inconsistency. Considered alongside the present findings, a sense of community belonging can coexist with doubts about particular judgments. Examining the psychological significance of community membership therefore requires attention to how members negotiate the relationship between group opinions and their own understanding.

Polarization experiences in open discussions were also associated with both psychological responses. When differing opinions enter the same discussion, students need to assess the grounds for each account, interpret the emotions expressed, and determine how these disagreements relate to their own understanding. From this perspective, making sense of and organizing diverse opinions remains a concrete cognitive task after exposure has occurred. Although online communities and open discussions provide different conditions for interaction, both involve group opinions serving as reference points for individuals’ understanding of issues. Examining the two forms of experience separately enables research on online group polarization to attend more closely to the questions and feelings students encounter when dealing with disagreement.

### 5.2. Cognitive and Emotional Responses in Relation to Judgment Formation and Reference Points Informing Choices

CDR was significantly and positively associated with VJD (β = 0.259, *p* < 0.001), suggesting that a need for cognitive adjustment can coexist with difficulty in judgment formation. When confronting contradictory opinions in a particular online discussion, students may have begun to reconsider their views while still searching for sufficient grounds to support a judgment. In this study, the response tendency encompassing discomfort arising from inconsistency, self-doubt, and adjustments in understanding was positively associated with subjective judgment difficulty. This suggests that students may still face unresolved questions as they reassess their existing understanding. Research on cognitive dissonance response would therefore benefit from examining how individuals address these questions and what information helps them establish a basis for judgment.

The positive association between CDR and VCS (β = 0.390, *p* < 0.001) connects cognitive adjustment to the adoption of evaluative reference points. Online opinions can challenge existing understanding while also providing material through which issues can be reconsidered. Opinion leaders’ interpretations of events and the emphasis on practical benefits in narratives of success may both become grounds for students to reconsider their judgments and choices. This finding suggests that understanding responses associated with cognitive dissonance also requires examining how students select new grounds for interpretation as they adjust their understanding. Reappraisal based on evidence and reliance on familiar external opinions in response to uncertainty are two circumstances that warrant distinction. Both may manifest as adjustments in understanding, but they involve different reasons and reference points.

ECR was significantly and positively associated with both VJD and VCS (β = 0.176, *p* = 0.001; β = 0.234, *p* < 0.001), indicating that the significance of emotional responses in online settings also extends to judgments about information and the bases for choices. Nabi (1999) explained the role of emotions in attitude formation through motivated attention and information processing. Following this account, descriptions of events in online discussions, together with others’ anger, anxiety, or sarcasm, may enter students’ interpretive processes and relate to which information they attend to and how they view conflicting explanations. The present findings provide empirical indications for examining these connections: as students respond to others’ emotions, they also develop an understanding of events and consider whether the accompanying opinions merit acceptance.

The associations of both cognitive and emotional responses with judgment formation and the reference points informing choices are consistent with the emphasis on cognitive and emotional media response states in the Differential Susceptibility to Media Effects Model (Valkenburg & Peter, 2013). In addition, after EPC, EPD, and the seven covariates were included, CDR and ECR retained a significant positive residual correlation (r = 0.556, *p* < 0.001), indicating shared variance that the current model did not explain. Future research could therefore track efforts to achieve cognitive consistency and emotional responses separately while also examining the contexts and individual states associated with both within the same interaction event. By including both psychological responses in the analysis of value-related judgments and choices, this study provides specific evidence of associations to inform this line of research.

### 5.3. A Process-Based Account of Value-Related Judgments and Choices

Understanding university students’ value-related judgments and choices requires attention to how they handle differences in understanding, respond to others’ emotions, and develop their own grounds for evaluation in online discussions.

After the two forms of polarization experience, the two psychological responses, and the seven covariates were included, VJD and VCS retained a significant positive residual correlation (r = 0.359, *p* < 0.001). This indicates shared variance not explained by the current regression equations. In evaluative activities, assessing whether a claim is credible is substantively connected to considering whether to use it as a basis for choices involving the public interest, consideration of practical benefits, or self-evaluation. The former involves understanding evidence, whereas the latter concerns the adoption of evaluative reference points. Placing both within a single framework facilitates further examination of how students move from understanding online opinions to articulating the reasons for their choices.

All four direct paths and eight specific indirect effects were statistically significant within the same model, providing a basis for considering experiences in interaction contexts alongside psychological responses. CDR and ECR help characterize the psychological associations connecting the two forms of polarization experience to judgments and choices. The direct associations retained for EPC and EPD indicate that these experiences also provide explanatory information beyond the two psychological responses. All predictors in the respective regression equations, including the seven covariates, explained 44.4% of the variance in VJD and 54.5% in VCS. These findings support considering students’ opinion environments together with their ways of responding when seeking to understand individual differences in value-related judgments and choices. The study’s contribution lies in specifying the relationship between online group polarization and university students’ values in terms of how they establish grounds for judgment, address doubts about their understanding, and adopt reference points informing choices. This provides an approach to examining how values are applied in online contexts.

### 5.4. Distinct Associations of Online Participation Patterns and Polarization Experiences

The covariate analysis suggests that time spent online and forms of participation should be examined separately. Online community membership status (Q7) and mode of participation in discussions of trending topics (Q9) were both positively correlated with VJD scale scores in the bivariate analysis (r = 0.089 and 0.101, respectively; both *p* < 0.01). After polarization experiences, psychological responses, and the other covariates were included simultaneously, their conditional associations with the VJD latent variable were negative (β = −0.082, *p* = 0.007; β = −0.096, *p* = 0.002). Average daily internet use, in contrast, had a positive conditional association with VJD (β = 0.058, *p* = 0.024). These results represent unadjusted correlations with scale scores and conditional associations within the current model, respectively, reflecting statistical relationships under different measurement and adjustment conditions.

This finding further distinguishes the information provided by participation behaviors from that provided by interaction experiences. Under the current coding and model specification, time spent online, community membership status, and mode of discussion participation displayed different patterns of association with judgment difficulty. The positive associations between both forms of polarization experience and judgment difficulty persisted after adjustment for these participation indicators. Students’ perceptions of specific opinion environments therefore provide information relevant to understanding judgment difficulty beyond time spent online and participation categories. Research on online experiences needs to consider both the behavioral context of participation and the subjective experiences accompanying it.

Familiarity with the issue and the availability of reference points for judgment are explanations for the negative conditional associations of Q7 and Q9 that warrant further testing. Students familiar with the relevant discussions may have accumulated more background information, or they may more readily develop a subjective sense of certainty. The former can support understanding of facts and evidence, whereas the latter may reduce perceived judgment difficulty. These possibilities have different implications for actual judgment quality. Future research could combine measures of participation experience with tasks involving source verification and evaluation of evidence to examine whether lower self-reported difficulty is accompanied by more adequate grounds for judgment. This would further clarify the relationships among participation patterns, interaction experiences, and actual judgment performance.

### 5.5. Practical Implications

Online literacy and values education in universities could integrate factual assessment, understanding of emotions, and consideration of competing values around a common issue. Classroom discussions of public events could ask students to compare conflicting accounts, verify sources, distinguish emotional expressions from the interests being advanced, and explain their reasons for accepting or revising a view. Activities addressing opinion leaders and narratives of success could further examine their evaluative standards, grounds for persuasion, and applicability to students’ own circumstances. These tasks correspond to the connections among cognitive responses, emotional responses, and evaluative reference points identified in this study.

Educational assessment could focus on source verification, the quality of argumentation, the weighing of interests, and the grounds for revising views. These assessments could be considered alongside students’ self-reported judgment difficulties and emotional experiences to identify where support is needed. Platforms could pilot clearer presentation of source information, issue background, and discussion context. The practical effectiveness of these suggestions remains to be tested through pilot courses or experiments involving specific interaction scenarios.

### 5.6. Limitations and Directions for Future Research

This study used cross-sectional self-report data from a single university, and the applicability of its conclusions needs to be extended through research across different institutions and student populations. The parallel mediation model identifies statistical indirect associations. Studies with multiple measurement occasions, contextual diaries, and experiments could help clarify the temporal ordering of cognitive and emotional responses, as well as possible reverse relationships between psychological responses and polarization experiences. Differences between cross-sectional indirect effects and parameters of longitudinal processes should also be considered in future designs (Maxwell & Cole, 2007).

Regarding measurement, EPC and EPD capture comprehensive subjective experiences, and adjustments in understanding within CDR may include informational influence. The general emotional responses captured by ECR and the persistence of emotion reflected in Q22 require further differentiation in relation to specific interaction events. AVE values for four constructs ranged from 0.406 to 0.495, leaving room for improvement in convergent validity. VJD overlaps in content with difficulty in evaluating information, while VCS encompasses the influence of online reference points across different evaluative domains. Future research could use issue-based tasks and actual choices to examine the scope of these measures and draw on process records to investigate specific stages of information processing. The two residual correlations may also reflect unmeasured psychological states, contextual factors, or common method components. Combining data from multiple sources with dynamic measurement would help distinguish these sources.

In addition, academic year and the internet use and participation variables were included as numeric covariates, implying linear relationships between their codes and the latent variables. Adjacent categories of community membership status and mode of discussion participation have different meanings, and the differences between them may not be equally spaced. The negative conditional associations of Q7 and Q9 with judgment difficulty therefore require further examination using categorical coding and measures of actual judgment performance.

## 6. Conclusions

Drawing on 2105 valid questionnaires, this study used samples of 770 and 1335 cases for exploratory analysis and confirmatory testing, respectively, to examine the associations between experience of online group polarization and university students’ value-related judgments and choices. After adjustment for seven covariates, both experienced polarization within online communities and experienced polarization in open online discussions were significantly and positively associated with cognitive dissonance response and emotional contagion response. Both psychological responses were also significantly and positively associated with value judgment difficulty and value choice susceptibility. All four direct paths and eight specific indirect effects were statistically significant, providing statistical support for the mediation hypotheses proposed in this study.

This study specifies the relationship between online group polarization and university students’ values in terms of judgment and choice processes. Cognitive adjustment can coexist with subjective judgment difficulty and is associated with the adoption of external evaluative reference points. The significance of emotional contagion response also extends to judgments about information and the bases for choices. Incorporating interaction contexts and both psychological responses within a single framework provides empirical evidence for understanding the doubts students encounter, the emotions they experience, and the evaluative reference points they use when engaging with online opinions. It also suggests directions for online literacy and values education in universities.

## Figures and Tables

**Table 1:** Variable definitions and measurement.

Latent Variable	Operational Definition	Items	No. of Items
Experienced polarization within online communities (EPC)	Subjective polarization experiences in online communities, encompassing community exposure, perceived homogeneity in content circulation, and discomfort and a tendency to defend the community when it is criticized.	Q10–Q12	3
Experienced polarization in open online discussions (EPD)	Subjective experiences of divisions in opinion, emotional expression, interaction rationality, and opposing positions in open discussion spaces, such as comment sections and on-screen comment streams (danmu).	Q13–Q15	3
Cognitive dissonance response (CDR)	Reported tendencies toward wavering judgments, discomfort arising from inconsistency, and adjustments in understanding when encountering online group opinions, with discomfort arising from inconsistency as the theoretical core.	Q16–Q18	3
Emotional contagion response (ECR)	Emotional responses during online arguments, the tendency to be influenced by others’ emotions, and the persistence of related emotions, with interpersonal emotional influence as the theoretical core.	Q20–Q22	3
Value judgment difficulty (VJD)	Subjective difficulty forming judgments about the veracity of online information, differing accounts, and complex issues.	Q23, Q24, Q26	3
Value choice susceptibility (VCS)	Self-reported susceptibility to online cues in choices involving the public interest, agreement with opinion leaders, consideration of practical benefits, and evaluations of oneself and one’s life.	Q29–Q31, Q34	4

Note: VJD and VCS scores respectively reflect respondents’ self-reported judgment difficulties and susceptibility to the influence of online cues. Scores are not assigned according to whether respondents’ value positions conform to a predetermined standard. Higher scores do not imply poorer judgment ability or “value deviation,” and lower scores do not imply more accurate judgments or superior values. Among the final 19 items, Q15 was reverse-scored to align its scoring direction with the other items; this scoring direction is unrelated to any evaluation of the quality of respondents’ values.

**Table 2:** Factor correlation matrix from the exploratory factor analysis.

Construct	EPC	EPD	CDR	ECR	VJD	VCS
EPC	1.000	—	—	—	—	—
EPD	0.246	1.000	—	—	—	—
CDR	0.301	0.058	1.000	—	—	—
ECR	0.362	0.142	0.577	1.000	—	—
VJD	0.300	0.254	0.435	0.466	1.000	—
VCS	0.337	0.040	0.469	0.479	0.442	1.000

Note: Factors were reordered to match the construct sequence, and their directions were aligned. Correlation coefficients were adjusted accordingly.

**Table 3:** Descriptive statistics and Pearson correlation matrix.

Variable	M	SD	Q1	Q2	Q4	Q5	Q7	Q8	Q9	EPC	EPD	CDR	ECR	VJD	VCS
Q1	0.49	0.50	1												
Q2	2.59	1.52	0.006	1											
Q4	0.28	0.45	0.285 **	0.047	1										
Q5	3.55	0.97	0.069 *	0.060 *	0.060 *	1									
Q7	2.65	1.39	0.099 **	−0.044	0.074 **	0.078 **	1								
Q8	3.12	1.19	0.028	−0.082 **	0.000	0.113 **	0.308 **	1							
Q9	3.13	0.97	0.049	−0.036	−0.003	0.091 **	0.306 **	0.362 **	1						
EPC	3.46	0.78	0.168 **	−0.015	0.097 **	0.183 **	0.360 **	0.325 **	0.360 **	1					
EPD	3.48	0.79	−0.076 **	−0.056 *	0.029	0.157 **	0.092 **	0.170 **	0.140 **	0.368 **	1				
CDR	2.93	0.79	0.047	−0.106 **	0.065 *	0.142 **	0.159 **	0.183 **	0.181 **	0.412 **	0.285 **	1			
ECR	2.89	0.98	0.106 **	−0.050	0.071 **	0.136 **	0.228 **	0.221 **	0.230 **	0.420 **	0.302 **	0.530 **	1		
VJD	3.41	0.85	0.058 *	−0.070 *	0.031	0.183 **	0.089 **	0.173 **	0.101 **	0.380 **	0.394 **	0.430 **	0.426 **	1	
VCS	3.06	0.67	0.016	−0.052	0.041	0.127 **	0.171 **	0.189 **	0.215 **	0.408 **	0.320 **	0.495 **	0.480 **	0.490 **	1

Note: * *p* < 0.05; ** *p* < 0.01.

**Table 4:** Reliability and Convergent Validity.

Variable	No. of Items	Cronbach’s α	CR	AVE
EPC	3	0.722	0.727	0.471
EPD	3	0.717	0.737	0.495
CDR	3	0.710	0.711	0.453
ECR	3	0.841	0.841	0.638
VJD	3	0.789	0.794	0.563
VCS	4	0.734	0.732	0.406

**Table 5:** Comparison of competing CFA models.

Model	Factor Structure	χ^2^	df	χ^2^/df	CFI	TLI	RMSEA	SRMR
Model 1	Six-factor model	509.029	137	3.72	0.951	0.939	0.045	0.042
Model 2	Five-factor model	945.380	142	6.66	0.894	0.872	0.065	0.052
Model 3	Four-factor model	1238.742	146	8.48	0.856	0.831	0.075	0.060
Model 4	Three-factor model	1574.811	149	10.57	0.812	0.784	0.085	0.065
Model 5	Two-factor model	2072.776	151	13.73	0.746	0.712	0.098	0.072
Model 6	Single-factor model	2416.414	152	15.90	0.701	0.663	0.106	0.077

**Table 6:** Structural model path estimates.

Hypothesis	Path	Standardized β	S.E.	z	*p*	Result
H1	EPC → CDR	0.508	0.068	7.426	<0.001	supported
H2	EPD → CDR	0.154	0.048	3.194	0.001	supported
H3	EPC → ECR	0.375	0.064	5.812	<0.001	supported
H4	EPD → ECR	0.183	0.044	4.121	<0.001	supported
H5a	CDR → VJD	0.259	0.059	4.380	<0.001	supported
H5b	CDR → VCS	0.390	0.064	6.061	<0.001	supported
H6a	ECR → VJD	0.176	0.053	3.341	0.001	supported
H6b	ECR → VCS	0.234	0.058	4.026	<0.001	supported
—	EPC → VJD	0.173	0.064	2.717	0.007	—
—	EPD → VJD	0.243	0.042	5.848	<0.001	—
—	EPC → VCS	0.155	0.061	2.526	0.012	—
—	EPD → VCS	0.103	0.040	2.592	0.010	—

Note: β, S.E., z, and *p* values were taken from the “STDYX Standardization” section of the Mplus output. The z values were taken directly from the reported Est./S.E. values and were not recalculated from rounded coefficients and standard errors. Values reported as *p* = 0.000 in the output are presented as *p* < 0.001. “—” indicates that no separate research hypothesis was proposed; all four of these paths were statistically significant.

**Table 7:** Covariate regression coefficients.

Covariate	EPC	EPD	CDR	ECR	VJD	VCS
Gender (Q1)	0.128 (<0.001)	−0.103 (0.001)	−0.034 (0.329)	0.045 (0.157)	0.026 (0.353)	−0.051 (0.082)
Academic year (Q2)	0.008 (0.778)	−0.070 (0.022)	−0.108 (<0.001)	−0.030 (0.261)	−0.023 (0.387)	0.009 (0.758)
Field of study (Q4)	0.047 (0.102)	0.046 (0.126)	0.040 (0.219)	0.016 (0.573)	−0.029 (0.279)	−0.010 (0.718)
Average daily internet use (Q5)	0.137 (<0.001)	0.177 (<0.001)	0.053 (0.072)	0.021 (0.440)	0.058 (0.024)	−0.003 (0.903)
Online community membership status (Q7)	0.260 (<0.001)	0.038 (0.234)	−0.047 (0.198)	0.040 (0.247)	−0.082 (0.007)	−0.011 (0.717)
Frequency of browsing discussions of trending topics (Q8)	0.190 (<0.001)	0.129 (<0.001)	0.001 (0.974)	0.034 (0.301)	0.028 (0.336)	−0.007 (0.805)
Mode of participation in discussions of trending topics (Q9)	0.256 (<0.001)	0.087 (0.009)	−0.021 (0.568)	0.041 (0.235)	−0.096 (0.002)	0.034 (0.291)

Note: *N* = 1335. Columns represent the latent variables being predicted, and rows represent the covariates. Cells report STDYX-standardized regression coefficients (β), with the corresponding two-tailed *p* values in parentheses.

**Table 8:** Bootstrap tests of indirect effects.

Hypothesis	Indirect Path	B	95% CI for B	β	S.E.	95% CI for β	*p* (β)	Result
H7	EPC → CDR → VJD	0.138	[0.072, 0.226]	0.132	0.036	[0.069, 0.211]	<0.001	supported
H7	EPD → CDR → VJD	0.046	[0.018, 0.088]	0.040	0.015	[0.015, 0.076]	0.008	supported
H7	EPC → CDR → VCS	0.162	[0.102, 0.245]	0.198	0.041	[0.128, 0.291]	<0.001	supported
H7	EPD → CDR → VCS	0.054	[0.020, 0.099]	0.060	0.022	[0.022, 0.109]	0.006	supported
H8	EPC → ECR → VJD	0.069	[0.028, 0.129]	0.066	0.024	[0.026, 0.119]	0.005	supported
H8	EPD → ECR → VJD	0.037	[0.015, 0.074]	0.032	0.012	[0.013, 0.063]	0.008	supported
H8	EPC → ECR → VCS	0.072	[0.034, 0.128]	0.088	0.027	[0.043, 0.149]	0.001	supported
H8	EPD → ECR → VCS	0.039	[0.017, 0.073]	0.043	0.015	[0.019, 0.078]	0.004	supported

Note: B denotes the unstandardized indirect effect, and β denotes the STDYX-standardized indirect effect; S.E. and *p* values correspond to β. Both sets of 95% confidence intervals are bias-corrected intervals obtained using 5000 bootstrap resamples.

## Data Availability

Data can be available on reasonable request from the authors.

## Abbreviations

The following abbreviations are used in this manuscript:

EPC	Experienced Polarization within Online Communities
EPD	Experienced Polarization in Open Online Discussions
CDR	Cognitive Dissonance Response
ECR	Emotional Contagion Response
VJD	Value Judgment Difficulty
VCS	Value Choice Susceptibility

## Appendix A

### Measurement Items Used in the Study

The questionnaire was administered anonymously. Participants were instructed to respond based on their actual online experiences and personal feelings during the previous six months. For Items Q10–Q34, responses were recorded on a five-point Likert scale ranging from 1 = Strongly disagree to 5 = Strongly agree.

**Table A1:** Measurement items used in the survey.

Construct	Item	English Wording	Coding
Experienced Polarization within Online Communities (EPC)	Q10	I often encounter online communities centered on specific interests, identities, or topics (e.g., gaming communities, fan groups, or communities for graduate-school entrance exam preparation).	—
	Q11	Within these communities, I feel that homogeneous content or viewpoints are repeatedly circulated.	—
	Q12	When others criticize an online community that I belong to, I feel uncomfortable and want to defend it.	—
Experienced Polarization in Open Online Discussions (EPD)	Q13	In comment sections or danmaku/bullet comments, I often see people’s opinions quickly split into two opposing camps.	—
	Q14	During online discussions, I often see emotionally charged comments (e.g., anger, ridicule, or anxiety).	—
	Q15	In discussions of trending events, I find that most internet users remain rational and do not readily take sides, report other users, or boycott them.	Reverse-coded
Cognitive Dissonance Response (CDR)	Q16	When I see many people online insisting on the same viewpoint, I begin to doubt my previous judgment.	—
	Q17	When the dominant opinion in an online group differs from what I truly think, I feel uncomfortable and conflicted.	—
	Q18	When faced with different viewpoints in online discussions, I repeatedly think them over and try to adjust my understanding.	—
Attention Check	Q19	This item is used to assess response attentiveness. Please select 2, “Disagree.”	Not included in scale scoring
Emotional Contagion Response (ECR)	Q20	Intense arguments online make me feel irritated, upset, angry, or distressed.	—
	Q21	When observing or participating in debates about trending topics, my emotions are easily influenced by the emotions of others.	—
	Q22	The bad mood caused by online arguments sometimes carries over into my offline life after I log off.	—
Value Judgment Difficulty (VJD)	Q23	I find it difficult to determine whether much of the information online is true or false.	—
	Q24	When people give different accounts of the same issue online, I find it difficult to judge who is right and who is wrong.	—
	Q26	When I encounter complex issues online that do not have a clear-cut good-or-bad answer, I find it difficult to make a judgment.	—
Value Choice Susceptibility (VCS)	Q29	Online discussions sometimes seem to speak only for a small group of people, which makes it difficult for me to make choices from the perspective of the public interest.	—
	Q30	I tend to agree with the views of online opinion leaders whom I like (e.g., influencers or content creators).	—
	Q31	After seeing many success stories online, I may give greater weight to practical benefits when making choices.	—
	Q34	Some trendy online expressions (e.g., songchigan [“a sense of ease”], neijuan [“involution”], and tangping [“lying flat”]) influence how I evaluate myself or my real-life circumstances.	—
Not included in scale scoring	Q25	Emotionally charged expressions online sometimes interfere with my rational judgment.	
	Q27	When some popular views online differ from my existing understanding of values, I feel confused.	
	Q28	When I encounter online content that questions mainstream values, I feel uncomfortable or even angry.	
	Q32	When participating in online discussions, I may use stronger language than I would in real life.	
	Q33	Online statements that disparage rationality and promote emotion can interfere with my rational acceptance of mainstream values.	

Note: Q19 was included as an attention-check item and was not used in the calculation of any construct score. Q15 was reverse-coded during data processing. After the questionnaire administration, through expert review, items Q25, Q27, Q28, Q32 and Q33 were excluded from the final scoring and model estimation.
